# Characterization of Photoactive Fe-TiO_2_ Lime Coatings for Building Protection: The Role of Iron Content

**DOI:** 10.3390/ma12111847

**Published:** 2019-06-06

**Authors:** Chrysi Kapridaki, Nikolaos Xynidis, Eleftheria Vazgiouraki, Nikolaos Kallithrakas-Kontos, Pagona Maravelaki-Kalaitzaki

**Affiliations:** 1 School of Architecture, Technical University of Crete, Polytechnioupolis, Akrotiri, 73100 Chania, Crete, Greece; xn1983@hotmail.com (N.X.); eltha07@gmail.com (E.V.); 2 School of Mineral Resources Engineering, Technical University of Crete, Polytechnioupolis, Akrotiri, 73100 Chania, Crete, Greece; kalli@mred.tuc.gr

**Keywords:** TiO_2_, Fe-doped TiO_2_, photocatalytic activity, Fe content, visible light, lime and Fe-TiO_2_ coating, self-cleaning, carbonation

## Abstract

Iron-doped TiO_2_ nanoparticles, ranging in Fe concentrations from 0.05 up to 1.00% w/w, were synthesized through a simple sol-gel method. Fourier-transform infrared spectroscopy (FTIR), X-ray powder diffraction (XRD), Ultraviolet-Visible (UV-Vis) spectroscopy, nitrogen adsorption−desorption isotherms, X-ray photoelectron spectroscopy (XPS), and X-ray absorption near-edge structure spectroscopy (XANES) were used to characterize the synthesized nanoparticles. The characterization of the Fe-doped TiO_2_ nanoparticles revealed the predominant presence of anatase crystalline form, as well as the incorporation of the Fe^3+^ ions into the crystal lattice of TiO_2_. The photocatalytic assessment of the Fe-doped TiO_2_ nanoparticles indicated that the low iron doping titania (0.05 and 0.10% w/w) have a positive effect on the photocatalytic degradation of Methyl Orange under visible radiation. Moreover, FTIR monitoring of calcium hydroxide pastes enriched with low Fe-doped TiO_2_ revealed enhancement of carbonation at both early and later stages. Improved photocatalytic performance and increased lime carbonation, observed in lime coatings with low Fe-doped TiO_2_ admixtures, established them as invaluable contributors to the protection of the built environment.

## 1. Introduction

The construction materials field is nowadays considered to be mainly responsible for the incremental activities in the energy sector associated with both consumption of energy resources and release of pollutants. The binders used in mortar and plaster technology, such as cement and lime, demand high energy to be produced, and the derived mortars applied to buildings consume energy resources for their maintenance. Effective binders in the design of building mortars can be considered those with a low number of additives, exhibiting self-cleaning features, hydrophobicity, water vapour permeability, and durability [1]. Nano-TiO_2_ is held in high regard in the construction sector, and is, therefore, incorporated in coatings, paints, plasters, and mortars in the cement industry. Its particular properties, ranging from the abatement of pollutants to the enhancement of fundamental mortar curing, such as carbonation and hydration, establish it as an additive of invaluable potentiality [1,2,3]. The photo-activation of TiO_2_ under UV irradiation was improved by shifting the semiconductor performance towards higher wavelengths, such as visible light, upon doping with metal and non-metal elements, thus imparting the doped-TiO_2_ self-cleaning features when incorporated into blends of construction materials [4]. Innovative solutions, intensively investigated so far, include research activities addressed to effectively activate TiO_2_ under visible light. Examples of well-performing building materials exhibiting multi-purpose aspects due to the effective combination of TiO_2_ with capable dopants were proposed previous studies [4,5]. 

Our research is addressed towards this direction. Having studied the specific features and durability of lime mortars and plasters in the conservation of historic masonry, the adopted scenario presented here included the synthesis of TiO_2_ doped with iron (Fe), its activation under visible irradiation, and further incorporation in lime coatings [4]. The choice of Fe is based on a multi-criterial decision, taking into account: (a) its presence in construction materials and the induced compatibility; (b) the low cost and its occurrence in a number of compounds; (c) its color, which could provide colored ochre surfaces; (d) its capability to be embedded into the TiO_2_ crystalline structure due to the similar radii of Ti^4+^ (0.68 Å) and Fe^3+^ (0.68 Å); and finally (e) the enhancement of the TiO_2_ photocatalytic activity, as Fe^3+^ ions play a crucial role in this procedure, functioning as hole and electron scavengers.

The effect of Fe nanoparticles on the enhancement of the TiO_2_ photoactivation and the influence of specific parameters, such as the content of iron ions, the titanium and iron oxidation state, structural data with crystallite size, textural and optical parameters of the various Fe-doped TiO_2_ nanoparticles, along with the way the Fe is embedded within the TiO_2_, were thoroughly studied. The Fe-doped nanoparticles were characterized by FTIR, XRD, UV-Vis, nitrogen adsorption−desorption isotherms, XPS, and XANES. The degradation of Methyl Orange (MO) was used as an indicator of the photocatalytic activity of the synthesized undoped and Fe-doped TiO_2_. Furthermore, TiO_2_ and Fe-TiO_2_ nanoparticles incorporated as additives in lime pastes were evaluated as promoters of carbonation.

## 2. Materials and Methods

### 2.1. Synthesis of Fe-TiO_2_ Nanoparticles and Pastes with Air Lime and Fe-TiO_2_

The photoactive Fe-doped nanoparticles were prepared by combining the sol-gel technique with thermal treatment and further calcination at 500 °C [6,7]. Titanium (IV) isopropoxide (TTIP, Sigma Aldrich, St. Louis, MI, USA) was used as a precursor for the TiO_2_ nanoparticles and Iron (III) nitrate nonahydrate (Fe(NO_3_)_3_ 9H_2_O, Panreac) as a Fe^+3^ dopant. Ethanol (EtOH, 99%, Sigma Aldrich, St. Louis, MI, USA) and deionized water were used as solvents, while sulphuric acid (H_2_SO_4_, Panreac, Barcelona, Spain) was used as a catalyst. Overall, five Fe-doped nanoparticles were synthesized, differing in the % amount of Fe^3+^. The doped synthesized TiO_2_ nanoparticles were denoted as §FeT, where the symbol § indicates the % (w/w) of Fe with respect to the TiO_2_ (0.05FeT, 0.10FeT, 0.20FeT, 0.70FeT, and 1.00FeT). For comparison purposes, undoped TiO_2_ nanoparticles, under the same experimental conditions, were also produced (0.00FeT). Table 1 lists the powders prepared with TiO_2_ and the different concentrations of iron ions. 

**Scheme 1:**
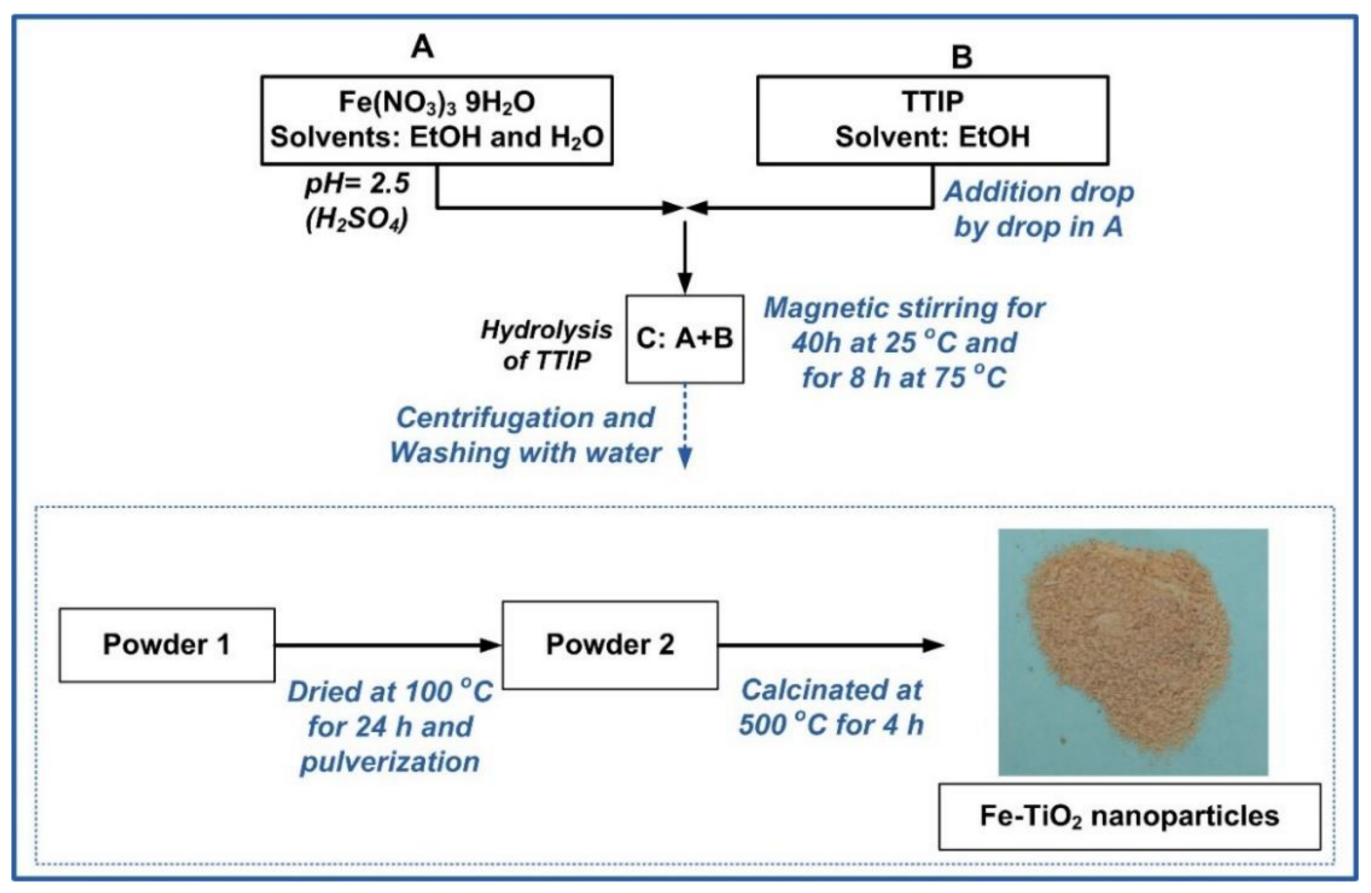
The experimental procedure for the preparation of Fe-TiO_2_ nanoparticles.

Scheme 1 illustrates in detail the whole experimental process used in this study. Specific care was taken in order to use a simple and non-laborious experimental procedure, guaranteeing the incorporation of Fe^3+^ into the TiO_2_ matrix and the production of anatase crystals under calcination consuming the minimum energy required. The molar ratio of the TTIP/H_2_O/EtOH was 0.03/2.30/1.56.

Firstly, the required amount of Fe(NO_3_)_3_·C9H_2_O was dissolved in EtOH and deionized water to produce solution A. The pH of this solution was adjusted to 2–3 using H_2_SO_4_ (1 M). The choice of H_2_SO_4_ relies on the promotion of the anatase phase during TTIP hydrolysis, as reported in several studies [8,9]. Next, TTIP diluted with EtOH, designated as B, was added into solution A drop-by-drop under magnetic stirring to provide solution C. Solution C was further subjected to magnetic stirring for 40 h at 25 °C and for 8 h at 75 °C, in order to facilitate both the hydrolysis of TTIP and the peptization process of the TiO_2_ nanoparticles. Next, the dispersed solution of TiO_2_ nanoparticles was centrifuged and washed with deionized water four times, so as for the final solid residue to be recovered (Powder 1). The latter was dried for 24 h at 100 °C and was pulverized to produce Powder 2. Finally, this powder was calcined for 4 h at 500 °C to provide the final Fe-doped TiO_2_ nanoparticles. The color of the designed nanoparticles was in direct relationship with the amount of the added Fe, ranging from slightly yellowish (0.05FeT) to brown (1.00FeT). The 0.00FeT nanoparticles (white color) were synthesized following the same procedure without adding the Fe(NO_3_)_3_·9H_2_O. 

The synthesized Fe-TiO_2_ nanoparticles were subsequently mixed with commercial calcium hydroxide (L: Lime, >96%, Honeywell Fluka, Fisher Scientific, Leicestershire, UK) at a quantity of 3% (w/w) to monitor the evolution of hardening through carbonation. For comparative purposes, pastes with lime and TiO_2_ nanoparticles (L0.00FeT), as well as pure lime (Blank), were also taken into consideration (Table 1). The synthesized pastes were casted into ceramic tubes of 3 cm in diameter and 1 cm in height. The above pastes were kept both in a curing chamber for setting at RH = 65 ± 3% and T = 20 ± 2 °C (19 h per day) and under sun light (5 h per day) for 45 days. 

### 2.2. Characterization of the Photoactive Fe-doped TiO_2_ Nanoparticles

Fourier Transform Infrared Spectroscopy (FTIR,) was used for the characterization of the chemical bonds of the synthesized TiO_2_ nanoparticles prior to calcination (Scheme 1, Powder 2). The absorption spectra were collected from a Perkin-Elmer 1000 spectrometer (Perkin-Elmer, Waltham, MA, USA) in the range of 400–4000 cm^−1^. The elaboration of the FTIR spectra was conducted with the aid of the Spectragryph software [10]. The FTIR analysis was carried out in the TiO_2_ nanoparticles before calcination in order to assess the hydrolysis and polymerization rate of TTIP. All the other analyses were conducted on the samples obtained after calcination at 500 °C. The crystalline phases of the calcined undoped and doped TiO_2_ nanoparticles were identified through the X-Ray Diffraction patterns recorded on a Brukerker D8 Advance diffractometer (Bruker, Billerica, MA, USA), operated at 35 kV and 35 mA with Cu Kα radiation with a nickel filter at a scan rate of 2° min^−1^ and a Bruker Lynx Eye strip silicon detector. The Brunauer, Emmett, and Teller (BET) surface area of the nanoparticles was determined by isothermal nitrogen adsorption-desorption at 77 K in a Micromeritics TRISTAR 3000 (TriStar, Norcross, GA, USA) and the outgassing of the samples was carried out via a VacPrep 061 (Micromeritics) apparatus ((TriStar, Norcross, GA, USA). The photoemission experiments were carried out in an ultra-high vacuum system (UHV) which consists of a fast entry specimen assembly, a sample preparation, and an analysis chamber. The base pressure in both chambers was 1 × 10^−9^ mbar. Unmonochromatized AlKα line at 1.486 × 10^3^ eV and an analyzer pass energy of 97 eV, giving a full width at half maximum (FWHM) of 1.7 eV for the Au 4f_7/2_ peak, were used in all XPS measurements. The XANES measurements were performed at the IAEA XRF beamline in Elettra Sincrotrone, Trieste, Italy [11], with Ring energy 2.0 GeV and Ring current 220 mA. XANES spectrometry was performed in an Ultra-high vacuum chamber (pressure 8.7 × 10^−9^ mbar) at total reflection X-ray fluorescence geometry, with a Silicon Drift Detector (SDD, Nano GmbH, XFlash 5030 Bruker, MA, USA), nominal resolution of 131 eV (at Mn-Kα). Spectra step was 0.2–2 eV (depending on the energy), with 2 repetitions, 5 s/step. The diffuse reflectance spectra (DRS) of the photoactive TiO_2_ nanoparticles were obtained using a UV-Vis Perkin-Elmer Lambda 35 spectrophotometer (Perkin-Elmer, Waltham, MA, USA) equipped with an integrating sphere Labsphere RSA-PE-20. The band gaps were calculated from the obtained Tauc plots. 

### 2.3. Assessment of the Photocatalytic Activity of the Photoactive Nanoparticles

The photocatalytic efficiency of the TiO_2_ nanoparticles was assessed by monitoring the degradation of the methyl orange compound (MO, Honeywell Fluka, Fisher Scientific, Leicestershire, UK) at room temperature under simulated Solar radiation. A solar radiation simulator (Model 96000, Newport, Owen, CA, USA) along with a 150-W xenon ozone-free lamp were utilized for the simulation of the solar radiation, which contains about 5% UV-A radiation and 0.1% UV-B irradiation, with a wavelength cut-off lower than 280 nm. In a typical experiment, the powdered photocatalysts were dispersed in an aqueous MO solution (5 ppm). For all the experiments carried out, the final concentration of the nanoparticles into the MO solution was equal to 0.8 g L^−1^. Prior to irradiation, the obtained dispersions were magnetically stirred in darkness for 30 min to attain an adsorption–desorption equilibrium between the dye and the photocatalysts. Next, the dispersions were subjected to the visible light at a distance of 10 cm, and this time was recorded as the onset of the photocatalytic experiment. It should be noted here that throughout the photocatalytic experiments the samples were magnetically stirred (360 rpm/min). Every 15 min, 4 mL of the irradiated solutions were collected with a pipette and the samples were centrifuged at 13200 rpm for 10 min. The maximum absorbance of the MO solution was measured using a UV–Vis Varian (Cary 1E) spectrophotometer (Varian Austalia Pty Ltd, Victoria, Australia) at the spectral range of 464–506 nm.

Taking into account that the photocatalytic degradation of MO can be ascribed to a pseudo-first order kinetic model, the recorded results revealed a good fit by non-linear regression (OriginLab) and the kinetic behavior of the nanoparticles followed Equation (1):

C = C_0_ e^−kt^, (1)

where C corresponds to MO concentration at various time intervals; k is the pseudo first order kinetic constant; t is the reaction time; and C_0_ is the MO concentration for t = 0. Both the values of k and the respective regression coefficients (r^2^) were acquired by means of this specific type of fitting. The r^2^ values obtained are indicative of a good fit between the experimental results and the model [12].

### 2.4. Evaluation of the Carbonation Process and Photocatalytic Performance of the Photoactive Fe-TiO_2_ Lime Pastes

The evolution of the carbonation process of the designed lime pastes with additives of Fe-TiO_2_ was monitored at pre-set time periods of 5, 15, 30, and 45 days. The powders were extracted from the core of the designed pastes, whose carbonation was interrupted after immersion in acetone; before the FTIR analysis, the extracted powders were dried at 70 °C for 30 h. 

The above-mentioned powders (100 mg) were dispersed in 20 mL of an aqueous solution of methylene blue (MB), with a concentration of 0.5 ppm. The same solutions containing lime and MB, as well as MB alone, were also used for comparison purposes. The degradation of MB was measured with a spectrophotometer (Konica Minolta Optics, Osaka, Japan) and the values of the total color differences (ΔE*) at a 7-day exposure under solar radiation were recorded. 

## 3. Results and Discussion

### 3.1. Characterization of the Photoactive Fe-doped TiO_2_ Nanoparticles with FTIR, XRD, BET, UV-Vis, and XPS

**Figure 1:**
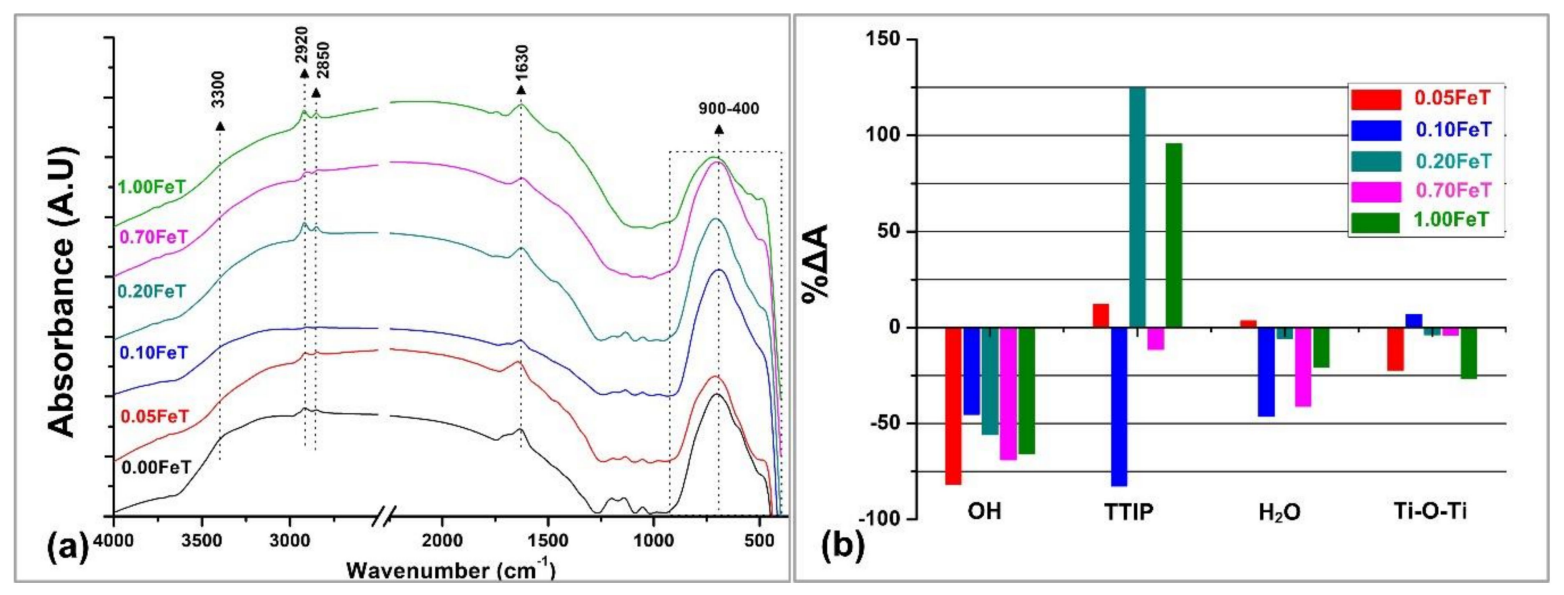
FTIR spectra (**a**) of the synthesized photoactive Fe-TiO_2_ nanoparticles along with the undoped TiO_2_ sample (0.00FeT) and (**b**) % difference of the integrated areas, ΔA: 3300 (OH), 2920–2850 (TTIP), 1630 (H_2_O), and 900–400 (TiO_2_) cm^−1^ compared with the 0.00FeT, according to Equation (2).

Figure 1a reports the FTIR spectra of the modified photocatalytic nanoparticles along with the spectrum of the undoped samples for comparison proposes, as obtained after drying and before calcination (Scheme 1: Powder 2). The spectra exhibited vibrations at the spectral ranges 3300, 2900, 1630, and 900 cm^−1^ were attributed to the O–H, C–H, H_2_O, as well as Ti–O and Fe–O bonds, respectively. Most importantly, the doublet at 2920 and 2850 cm^−1^, which is evident in all the spectra except for the 0.10FeT, originate from the –CH_2_ and –CH_3_ of TTIP [13]. The presence of –CH_2_ and –CH_3_ evidenced the partial hydrolysis of TTIP; the latter further influences the amount of the active TiO_2_ in the powders. On the other hand, the wide band ranging from 900 to 400 cm^−1^, observed in all of the FTIR spectra, assigned to the chemical bonds of Ti–O, Ti–O–Fe, and Fe–O, evidenced that the hydrolysis of the major part of TTIP and the subsequent formation of TiO_2_ nanoparticles were achieved [14,15,16]. Comparing the Fe-TiO_2_ spectra with the undoped TiO_2_ (0.00FeT) spectrum, the observed changes in the intensity and the redshift of the band 900–400 cm^−1^ can be interpreted as a contribution of the new-formed Fe/TiO_2_ system [15,16]. Furthermore, the peaks that appeared at 1130, 1199, and 1050 cm^−1^ can be ascribed to the S–O and S=O bonds derived from H_2_SO_4_ residues used for the pH adjustment during the sol-gel process [17]. Finally, the shoulder in the spectral region 3000–3500 cm^−1^ is related to the stretching modes of surface water molecules or to the hydrogen-bonded surface OH groups. These OH groups in combination with the absorbed water (bending mode of water molecules at 1630 cm^−1^) may positively contribute to the photocatalytic activity of the TiO_2_ nanoparticles, functioning as hole-scavengers [18]. Although the FTIR spectrum of water is complex, the bending of the –OH groups at 1630 cm^−1^ is a characteristic absorption, as it is sharp, intensive, and does not overlap with the peaks of other groups. 

In order to quantify the spectral differences of Figure 1a, the integrated areas (A) of the significant peaks were estimated at the spectral ranges of: (a) 3500–3000 cm^−1^, corresponding to the OH groups, (b) 2920–2850 cm^−1^, corresponding to the unhydrolyzed TTIP, (c) 1630 cm^−1^, indicating the absorbed water and the hydrophilicity of nano-particles, and (d) 900–400 cm^−1^, indicating the Fe/TiO_2_ formation (Figure 1b). All the aforementioned parameters were correlated to those of the undoped TiO_2_ (0.00FeT), according to Equation (2):

(2) $$\%\;\Delta A=\frac{FeTsample-0.00FeT}{0.00FeT}\times 100,$$

The graph of Figure 1b revealed that the 0.10FeT exhibited a high negative difference for the TTIP, indicating effective hydrolysis. This was also corroborated by the positive value in the Ti-O-Ti formation for the 0.10FeT related to the production of TiO_2_. On the other hand, the 0.20FeT and 1.00FeT presented the lowest hydrolysis of TTIP. In addition, the doped samples absorb less water than the undoped TiO_2_, as indicated by the lower values of OH and H_2_O, with the exception of the 0.05FeT, exhibiting a slightly higher value in the water absorption than the undoped sample. However, the 0.05FeT along with the 1.00FeT exhibited the worst performance in the Ti-O-Ti formation, which plays an important role in the photocatalysis of TiO_2_. 

**Figure 2:**
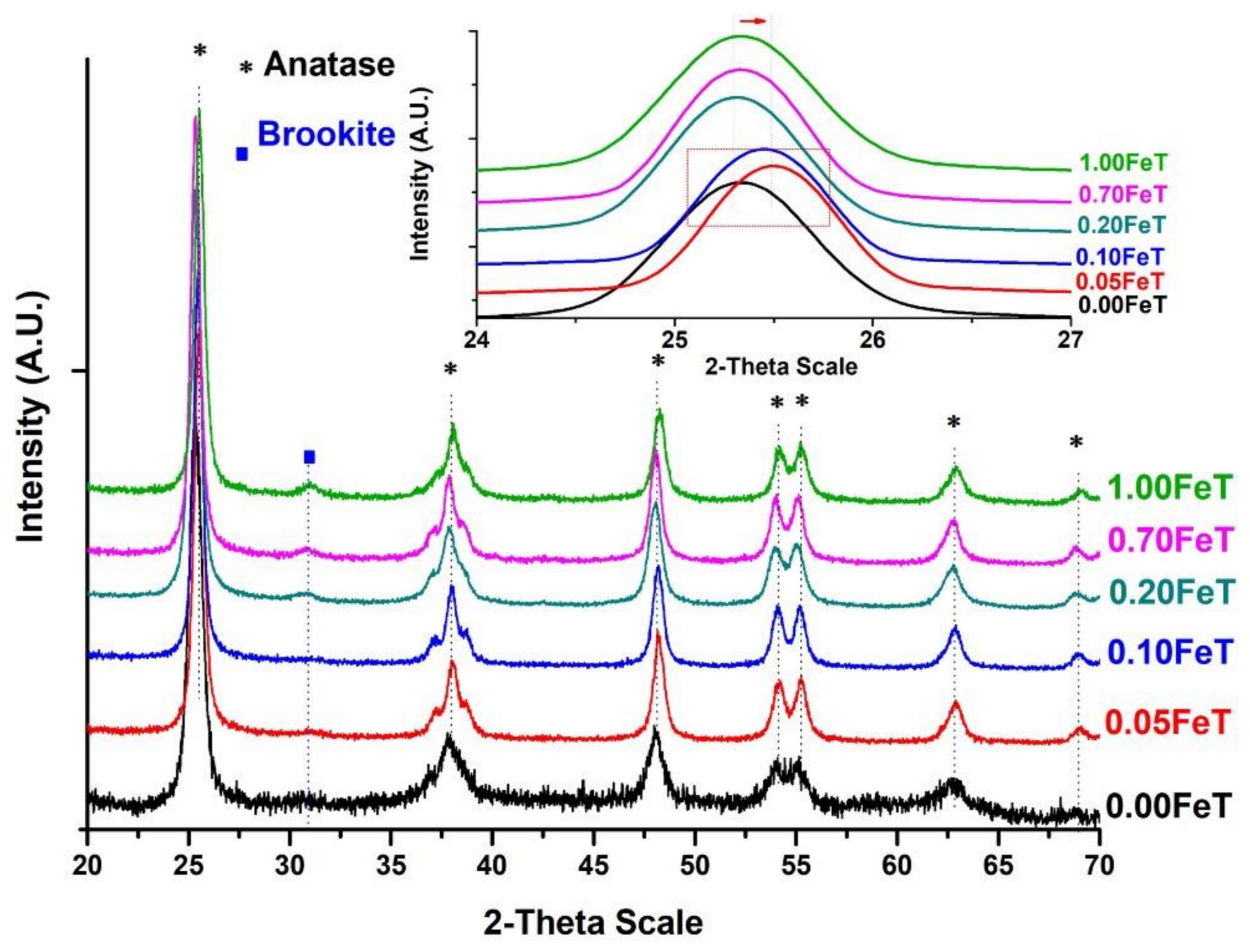
X-rays diffractograms of the doped and undoped TiO_2_.

The XRD results of the Fe-doped synthesized nanoparticles, presented in Figure 2 and Table 2, revealed that the major crystalline phase of TiO_2_ is anatase (25.3°, 37.8° and 47.9°), accompanied by the secondary phase of brookite (31.2°) [14,19]. No diffraction peaks corresponding to secondary Fe_2_O_3_ phases were detected, most probably due to both the homogeneous distribution of Fe^3+^ into the titania host lattice, replacing the Ti^4+^ ions, and the low quantity of Fe [4,6,20,21].

According to the XRD results of Figure 2 and Table 2, the percentage of the brookite phase is associated with the concentration of Fe, as this crystalline phase was not observed in the undoped nanoparticles. The increment of the brookite is directly related to the synthesis environment of the TiO_2_ nanoparticles and is promoted under acid conditions (pH<2.5), even at low annealing temperatures [20,21]. Moreover, it has already been established that the incorporation of Fe into the TiO_2_ crystal lattice induces arrangement of the tetragonal structure of the anatase phase to the orthorhombic phase of brookite [14]. Therefore, the presence of brookite is directly correlated with the replacement of the Ti^4+^ ions from Fe^3+^ within the anatase lattice [14]. In the cases of 0.05FeT and 0.10FeT, these further support the embedding of Fe^3+^ into the TiO_2_ crystal structure derived from the shift of the main peak of anatase from 25.30° to 25.50°, as is clearly shown in the inset of Figure 2 [4]. The crystallite size of anatase decreased in the Fe-doped samples, in accordance with the bibliographic data, due to the defects induced in the crystal lattice upon the incorporation of Fe^3+^ into the anatase, not allowing its growth [14]. The doped TiO_2_ nanoparticles showed an increasing surface area comparing to the undoped TiO_2_, but this feature is not in direct relationship with the Fe content.

The band gaps of the TiO_2_ nanoparticles calculated from the diffuse reflactance spectra using the Tauc plots are presented in Table 2 [12]. The band gap of the undoped TiO_2_ reduced from 3.13 eV to 2.61 eV for Fe-doped samples. As expected, a clear relationship exists between energy band reduction and Fe content increase, attributed to the charge-transfer transition between the Fe-d electrons and the conduction band of TiO_2_ [16]. 

**Figure 3:**
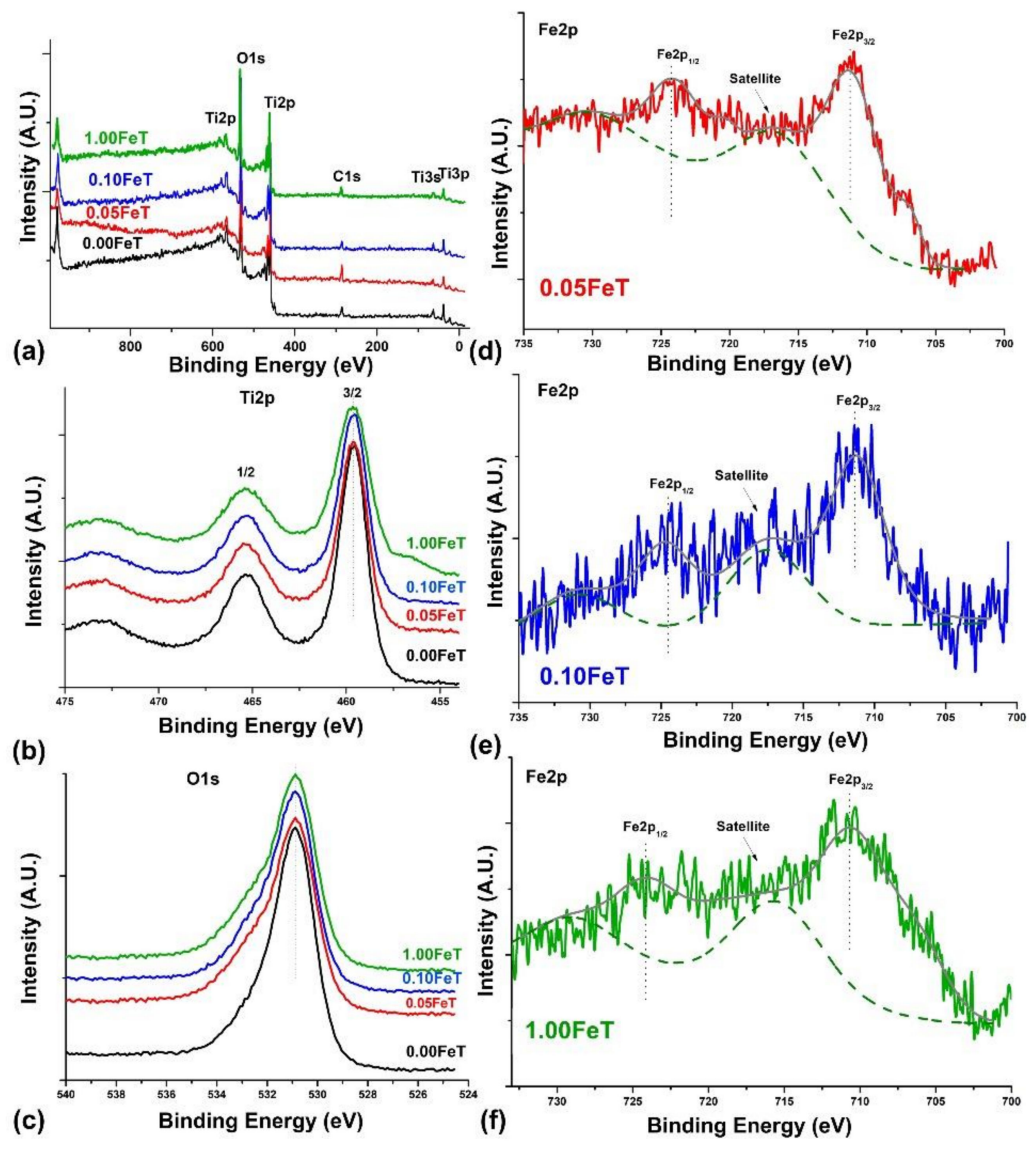
Wide-survey XPS spectra (**a**) and high resolution XPS spectra of Ti 2p (**b**), O 1s (**c**), and Fe 2p (**d**–**f**) of the undoped (0.00FeT) and Fe-doped nanoparticles (0.05FeT, 0.10FeT, and 1.00FeT).

In order to gain further insight into the crystal lattice of the synthesized TiO_2_ nanoparticles, XPS analysis was performed on the samples with low Fe content, such as 0.05FeT and 0.10FeT, the sample with the highest Fe content (1.00FeT), and the undoped TiO_2_ (0.00FeT). Figure 3 illustrates the obtained XPS results of Fe-doped and undoped nanoparticles. The wide-survey spectra (Figure 3a) of the synthesized nanoparticles showed all the predominant peaks of the samples. However, for the needs of this study, the spectra of Ti 2p and O 1s are separately depicted in Figure 3b,c, respectively.

In the Ti 2p core level spectra of all the nanoparticles, two distinct peaks appeared (Ti 2p_3/2_ at 459.5 eV and Ti 2p_1/2_ at 465.4 eV), showing that the oxidation state of titanium is Ti^4+^ (Figure 3b) [22]. Both the symmetry of these peaks and the absence of shoulders are correlated with the formation of TiO_2_ crystals without peculiar defects [22]. The peak of O 1s at 530.9 eV (Figure 3c), attributed to TiO_2_, exhibit an asymmetry to higher binding energies (~ 533 eV), due to the presence of OH groups on TiO_2_ surface [23,24]. The latter is in accordance with the FTIR results, indicating the presence of the hydroxyl bonds. By comparing the peaks of Ti 2p and O 1s of the Fe-doped samples with the undoped sample, no shifting of the peaks in the doped samples occurred, but only a widening as a function of the Fe increasing content. These results may be related to a Fe ion embedment into the crystal lattice [24]. Finally, the Fe 2p XPS superimposed spectra of the Fe-TiO_2_ with Fe 2p_3/2_ and Fe 2p_1/2_ peaks at 711.2 and 724.1 eV, respectively, (Figure 3d–f), demonstrated that Fe mainly existed in the +3 oxidation state [22]. In addition, the satellite peak located at 717.2 eV further supported the presence of the Fe^3+^ ionic state [23]. Taking into account the obtained oxidation states of titanium (Ti^4+^) and iron (Fe^3+^), it can be concluded that the Fe^3+^ ions are indeed incorporated into the TiO_2_ crystal lattice through the replacement of Ti^4+^ ions. As has already been mentioned, the replacement of Ti^4+^ ions from Fe^3+^ can be achieved because of their similar radii and electronegativity.

### 3.2. XANES Analysis of Samples

XANES can be used as a fingerprint of the chemical environment of the probed element. The intensity, shape, and position of the pre-edge features in a XANES spectrum reflect the redox state, hybridization, and symmetry of the probed element sites [25,26]. These pre-edge features, associated with the coordination number of a central atom, have been utilized as a powerful fingerprint for the coordination sphere in various samples; nevertheless, the simple utilization of the pre-edge peak height is not always adequate to evaluate the coordination number, because the peak intensity differs remarkably from the coordination symmetry, even at the same coordination number [27]. Therefore, incorrect interpretations about pre-edge peaks can be deduced, especially in cases of using XANES spectroscopy as a unique tool for sample characterization. Consequently, XANES spectroscopy has to be combined with other spectroscopic techniques in order for the geometry and coordination of the probed element to be revealed. 

**Figure 4:**
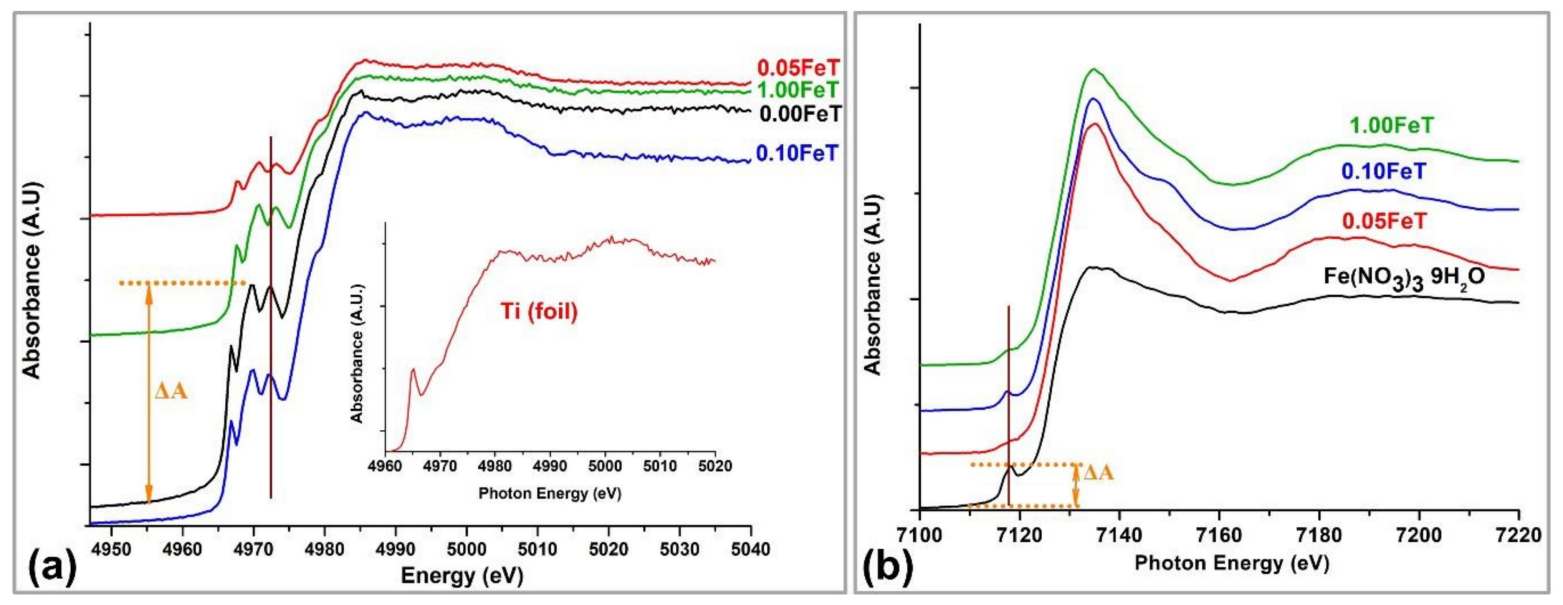
Ti K-edge (**a**) and Fe K-edge (**b**) XANES spectra for 0.00FeT, 0.05FeT, 0.10FeT, and 1.00FeT nanoparticles. XANES spectra of Ti foil and Fe(NO_3_)_3_ 9H_2_O were used as reference samples. The brown cursors and yellow arrows indicate the pre-edge peaks position and height, respectively, of the reference samples.

The oxidation state, coordination number, and centrosymmetry of Ti and Fe were further investigated through the study of the Ti and Fe K-edges XANES spectra, illustrated in Figure 4. For comparison purposes, the Ti K-edge spectrum of a Ti metal foil sample is shown as an inset in Figure 4a. All the pre-edge features of the spectra illustrated in Figure 4 present low intensities, as they originate from dipole forbidden transitions to unoccupied orbitals [25]. 

At first sight, the Ti XANES spectra illustrated in Figure 4a pointed out that the Fe-TiO_2_ exhibit similar features in both pre-edge and post-edge areas. Specifically, in the pre-edge area of Ti K-edge XANES spectra, three distinguished peaks were observed. In the case of the Ti K-edge, these three pre-peaks were derived from the hybridization of p and d orbitals of the Ti and its surrounding atoms [28]. The slight shoulder and the main peak of the curve that appeared in all of the Ti spectra are associated with the dipole-facilitated transition 1s → 4p [28]. 

The 0.10FeT sample exhibited similar spectral features, such as number, pre-edge heights, and main peak height with the undoped TiO_2_ (0.00FeT) (Figure 4a). According to published data, the spectrum of anatase, being the only crystalline form identified in the undoped TiO_2_, corresponds to a sixfold coordination [26]. Based on the similarity of spectra, it can be assumed that the coordination number of Ti in the 0.10FeT sample is close enough to that of the 0.00FeT sample. 

It has been recognized that the intensity of the pre-edge peaks increases significantly with the decrease in coordination number and enhancement of the hybridization degree, and is associated with an increased distortion of the centrosymmetric structure [25,26,27,29]. Therefore, it was deemed important to compare the height of the second peak (ΔA) of the Fe-doped samples with the undoped one (Figure 4a). The resulted fraction follows the decreasing order: 0.677 (for 0.10FeT), 0.581 (for 1.00FeT), and 0.231 (for 0.05FeT). According to the derived results, we can conclude that Ti in the samples 0.10FeT and 1.00FeT may exist in a coordination number slightly higher than six, but with an enhanced orbital p-d mixing and hybridization into the crystal lattice. Besides, Ti in the 0.10FeT seems to participate in a more distorted octahedral structure than in all the other examined samples, due to the higher intensity of this pre-edge peak compared to the other doped samples. 

Another observation was derived from the position of the three main pre-edge peaks in Figure 4a, where the first one is indicated by the cursor. It is known that the displacement of the pre-edge peaks to higher energy reveals an enhancement of the oxidation state for the probed element [25]. Under this consideration it can be concluded that the oxidation state of Ti in 0.10FeT is equal to 4+, whereas in the case of 0.05FeT and 1.00FeT the oxidation state seems to be slightly increased.

Figure 4b demonstrates the Κ-edge XANES spectra of the Fe-doped nanoparticles, along with the spectrum of Fe(NO_3_)_3_ 9H_2_O for comparative purposes. The pre-edge peak is associated with the forbidden transition from 1s to 3d, which is eventually partially allowed, because of the interaction between the d orbitals of the metal and the p orbitals of its surrounding oxygen atoms [30]. The width-shape and height of this peak depend on the surrounding geometry and coordination number of the Fe atoms, while the Fe oxidation state depends on the energy shift [25,31]. Most importantly, the intensity of the pre-edge features reflects the degree of the distortion of centrosymmetry [31,32]. The higher the pre-edge peak is, the larger the deviation from centrosymmetry becomes. This criterion can be applied to our data concerning the Fe-XANES spectra, as it has also been applied in the case of Ti. The maximum deviation from centrosymmetry is observed for the 0.10FeT and the smallest for 0.05FeT, while the 1.00FeT shows intermediate structure. This consideration concerning the deviation from the centrosymmety for the doped samples is in agreement with that derived from the K-edge Ti XANES spectrum, indicating that Fe and Ti participate in a distorted octahedral structure. 

Figure 4b shows that the position of the pre-edge peak varies at the different Fe spectra, while it is almost absent in the case of the Fe0.05T sample. Given that the pre-edge shift reflects the oxidation state of the probed element, the first derivative was applied to the studied spectra in order to find precisely the pre-edge peak position. The latter indicated that the average oxidation state of Fe in the 0.10FeT is close enough to that of the Fe(NO_3_)_3_ 9H_2_O, followed by 0.05FeT and 1.00FeT. 

In order to further verify the oxidation state of Fe in the examined samples, Linear Combination Fitting (LCF) analysis was employed. LCF is a tool that identifies and quantifies the different chemical species that might coexist in an examined sample [33,34,35]. LCF analysis makes linear combinations of reference spectra and fits them to the experimental data in order to yield the least fit to the spectrum of the investigated sample. As reference samples, a metal Fe foil, siderite (FeCO_3_), Fe(NO_3_)_3_ 9H_2_O, franklinite (ZnFe^3+^_2_O_4_), and magnetite (Fe_3_O_4_) were used in order to simulate Fe in different oxidation states, ranging from 0 to 3+. The results revealed that Fe^0^ did not exist in any of the examined samples, while Fe^3+^ and Fe^2+^ coexist. More specifically, the 0.10FeT, 0.05FeT, and 1.00FeT contained 90.3%, 85.6%, and 83.0% Fe^3+^ (w/w), respectively. 

A slight disagreement was observed when comparing XPS and XANES spectroscopic methods. This is due to the fact that XANES is a bulk technique and provides information about the average oxidative state of the probed element in a volume sample. In conclusion, the mean oxidation state of Ti in undoped TiO_2_ and 0.10FeT is equal to 4+, while the 0.05FeT and 1.00FeT showed a slightly higher oxidation state of Ti. The Fe exhibited the higher Fe^3+^ content in 0.10FeT, followed by 0.05FeT and 1.00FeT. Both Fe and Ti XANES spectra of 0.10FeT indicate a greater distortion from the centrosymmetry compared to the other samples, directly correlated to the better embedding of Fe into the TiO_2_ structure.

### 3.3. Photocatalytic Activity of the Fe-doped TiO_2_ Nanoparticles

**Figure 5:**
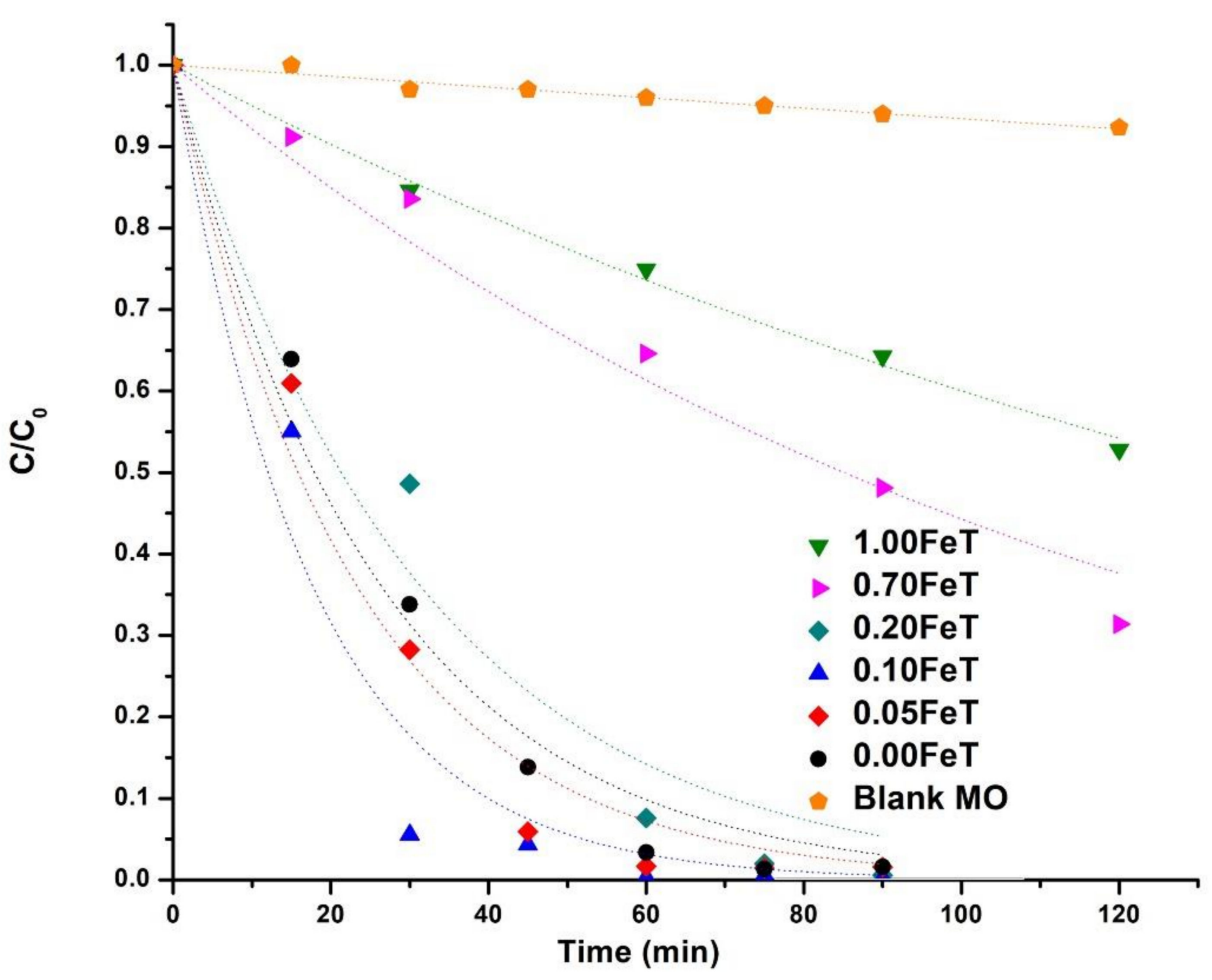
Photocatalytic efficiency of the synthesized nanoparticles.

The photocatalytic activity of all of the synthesized TiO_2_ nanoparticles was evaluated by monitoring the degradation of MO under solar lamps (Figure 5 and Table 3). The photodegradation of MO was pseudo-first order rate and the constants (k) along with the regression coefficients (r^2^) are listed in Table 3 [36]. It can be seen from the plots of Figure 5 that the photochemical degradation of MO (Blank MO) is negligible. Comparing the photocatalytic efficiency of the nanoparticles, the lowest activity was clearly recorded for the 1.00FeT (k = 5 × 10^−3^ min^−1^), while the maximum activity was derived from the 0.10FeT sample (k = 58 × 10^−3^ min^−1^). The 0.05FeT and 0.00FeT samples show similar behavior, nevertheless, it should be noted at this point that the 0.10FeT induced the maximum degradation of MO within 30 min (94%), followed by 0.05FeT with 71% and 0.00FeT with 66% (Table 3). As it has already been mentioned, the analyses of the nanoparticles through the XRD, XPS, and XANES techniques elucidated that the major Fe content existed in Fe^3+^ oxidation state and it has been successfully incorporated into the TiO_2_ crystal. Therefore, the photocatalytic results revealed that the Fe ions in the nanoparticles in low concentrations, such as 0.05% and 0.10% w/w, improved the photocatalysis, establishing those Fe contents as the optimal dopants in TiO_2_. 

It is well known that the enhancement of the Fe-doped TiO_2_ photocatalytic activity under visible light is due to both the separation of the hole-electron pair and the photogeneration of additional electrons in the TiO_2_ conduction band [37]. More specifically, Fe^3+^ ions can function as hole and electron scavengers, thus inhibiting the recombination of the photogenerated charge carriers and producing Fe^2+^/Fe^4+^ ions [38,39]. The latter ions are unstable compared to Fe^3+^, owing to their half-filled 3d^5^ orbital; therefore, they have the tendency to react with the absorbed O_2_ or the –OH groups of the neighboring surface Ti^4+^ ions, playing an active role in the photocatalytic decomposition of MO [37]. Moreover, the contribution of the t2g level of the 3d orbital of Fe^3+^ to the photocatalytic procedure under visible light is based on the absorption of a photon by the metal ion and the transfer of an electron to the conduction band of TiO_2_ [38]. The produced electron and Fe^4+^ ions further react with the absorbed O_2_ and the hydroxyl group, respectively, thus additionally promoting photocatalysis [38,39]. However, it should be mentioned that the beneficial role of the Fe^3+^ ions as hole and electron scavengers can be achieved up to an optimum Fe concentration, beyond which the Fe^3+^ ions act as recombination centers, inducing decline of the photocatalytic activity [37,38]. An example sustaining this fact is derived, in our case, from the synthesized 1.00FeT nanoparticles, which induce remarkable reduction of the photocatalytic activity compared to 0.05FeT and 0.10FeT, because of this reverse function of the Fe^3+^ ions (see Figure 5). 

### 3.4. Assessment of the Carbonation Process and the Self-Cleaning Properties of the Lime Pastes

**Figure 6:**
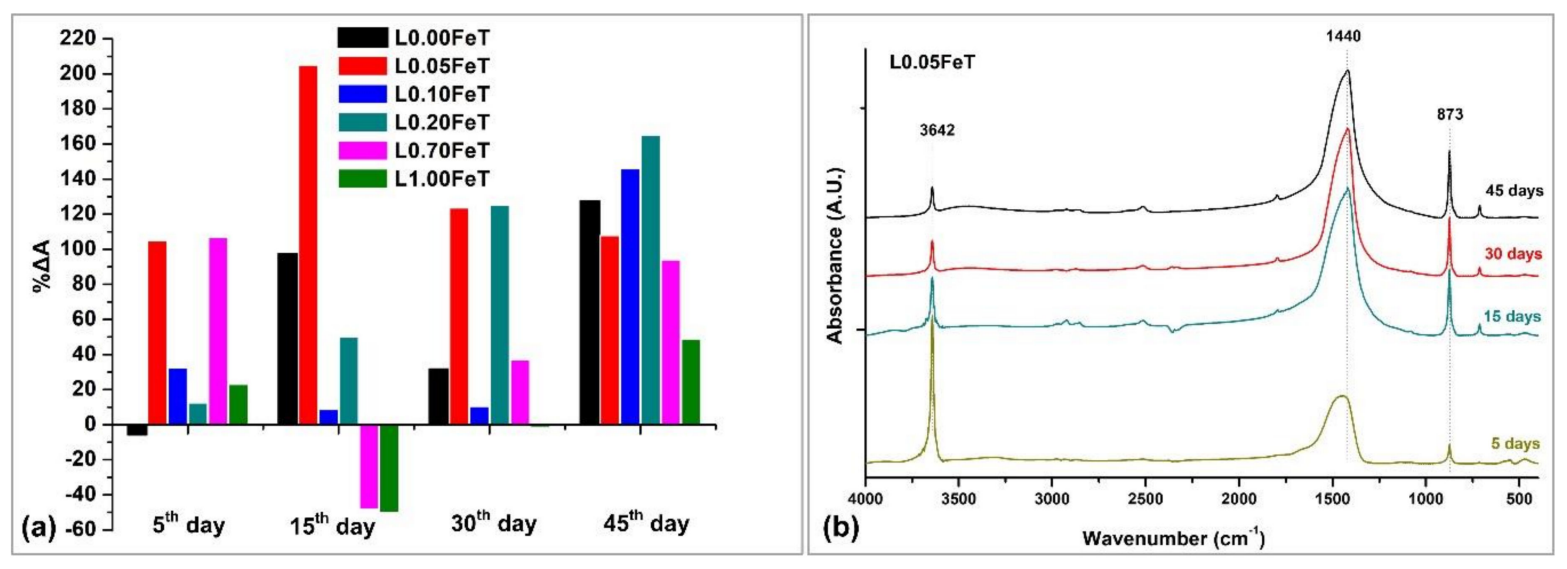
Evolution of the carbonation process of the pastes (**a**) and FTIR spectra of L0.05FeT as a function of the curing time (**b**).

The assessment of the evolution of carbonation in lime pastes with admixture of iron doping titania was carried out through FTIR analysis and the results are depicted in Figure 6. The evolution of the carbonation process was obtained by monitoring the ratio of the intensity of the height of peaks corresponding to the neo-formed calcite (873 cm^−1^) and portlandite (3642 cm^−1^) versus established curing time of the lime pastes (Table 1 and Figure 6a). The peak of calcite increased, whereas the peak of Ca(OH)_2_ was reduced over curing time. 

Enhanced carbonation was recorded for the Fe-TiO_2_ lime pastes in early curing time (5 days) compared to the pure lime paste, as opposed to the undoped TiO_2_ lime sample showing a negative value (Figure 6a). However, in previous work TiO_2_ enhanced lime carbonation, even in early curing stages, under UV irradiation [1]. In this study, after 5 days under visible irradiation, only the doped-TiO_2_ admixture affected the carbonation of lime positively, while the TiO_2_ lime required more time in order for the carbonation to be promoted. This information demonstrated the beneficial effect of Fe-TiO_2_ admixture in external lime coatings, which can carbonate, and therefore, harden faster in the early stages under visible light. Despite the increasing carbonation on a 5-day exposure to solar light of the doped samples, a clear relationship was not registered between evolution of carbonation and photocatalytic activity of the studied admixtures at all the measured stages. As an example, the least photocatalytically performing nanoparticles, namely 0.70FeT and 1.00FeT, when added to lime coatings retarded the carbonation on the 15th day and promoted it later. On the other hand, it was expected that the best performing photocatalysts, such as 0.01FeT and 0.05FeT, would enhance the carbonation of lime pastes at all the stages. However, only the 0.05FeT fulfils this assumption, whereas the most effective photocatalyst, 0.10FeT, induced a high carbonation level in the lime paste only after 45 days of curing.

The L0.05FeT paste, which performed best at all the measured stages, showed a peak with a twofold carbonation at 15 days, far surpassing the performance of all the studied formulations. Therefore, in Figure 6b indicative FTIR spectra of L0.05FeT for specific curing time are displayed, evidencing the evolution of carbonation over time by decreasing the portlandite at 3642 cm^−1^ and increasing the sharpness of calcite peaks at 1440 and 873 cm^−1^. 

In an attempt to explain the results obtained on the evolution of carbonation under visible irradiation, it should be noted that carbonation is a dynamic process conferring continuous change to the microstructure [40,41]. The calcium carbonate deposited on the surface pores may function as an obstacle for the CO_2_ diffusion inside, thus preventing achievement of full carbonation. This is the reason for the reduced carbonation observed in the sample L0.10FeT containing the best photocatalyst, where more CO_2_ is expected to be produced due to the advanced photocatalysis, thus contributing to the formation of a calcite surface layer further preventing the CO_2_ diffusion inside. A supporting finding for this statement is the calcite identification on the surface layer in an abundant amount and at a minor amount at a depth of 2 mm from the surface by FTIR. 

**Figure 7:**
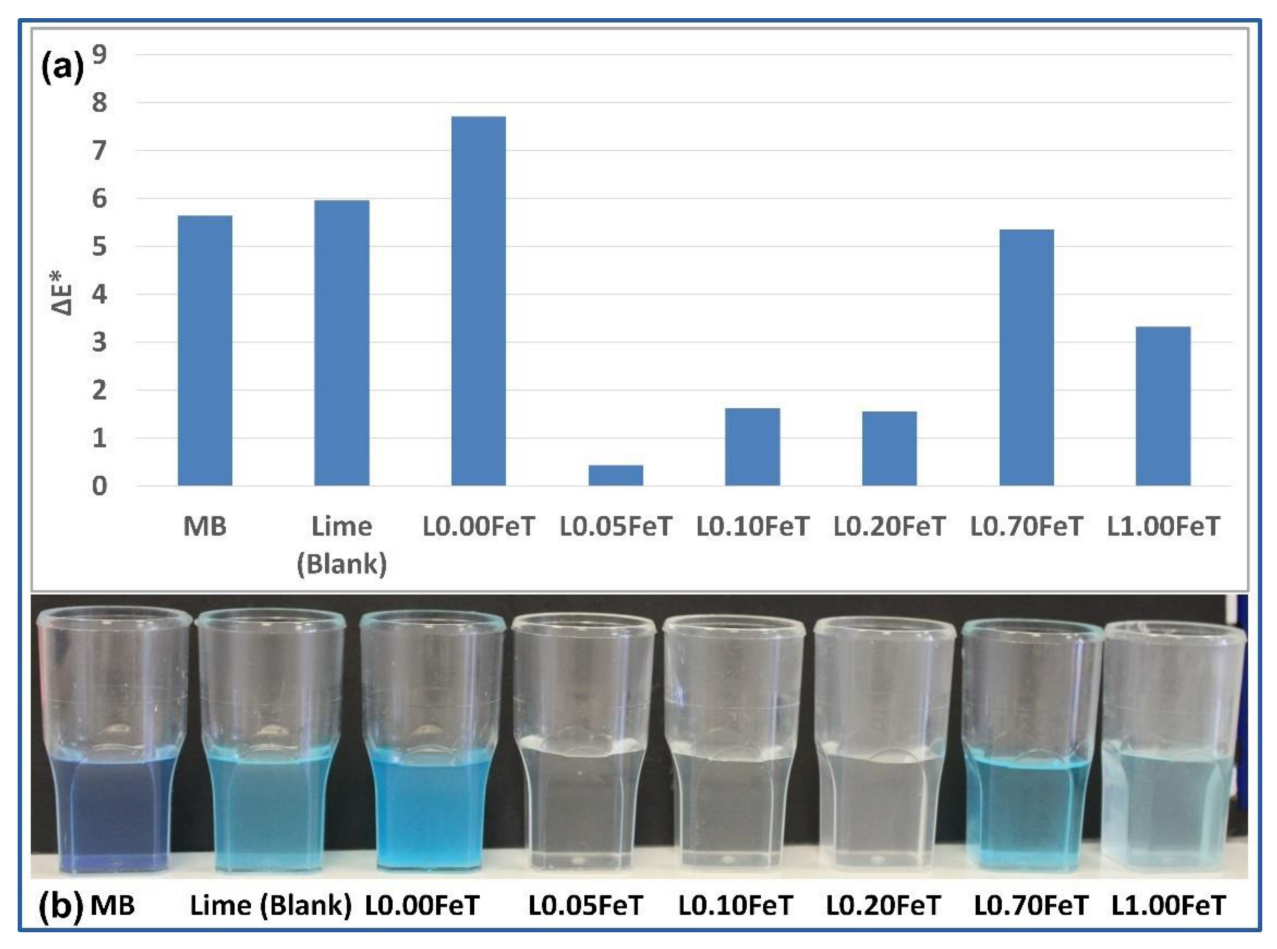
Total color difference ΔE* of methylene blue (MB) in lime pastes containing doped and undoped TiO_2_ (**a**) and the corresponding images of samples after 7 days under solar irradiation (**b**).

The photocatalytic experiment of the TiO_2_-enriched lime pastes subjected to solar radiation for 7 days is illustrated in Figure 7 and compared with samples of MB and lime (blank) without photocatalysts. As can be appreciated from Figure 7b, the best performing samples are those with low iron content, precisely up to 0.20% w/w. The ΔE* total color difference, measured with deionized water as a reference sample, presents the lowest value for the L0.05FeT sample, in line with the best observed carbonation, followed by the samples L0.10FeT and L0.20FeT. These results demonstrate again the significant role of the iron content in the doping of TiO_2_, by first degrading the pollutant and secondly enhancing the carbonation of lime. 

Taking into account the results of all the experimental data, it can be concluded that the 0.05FeT sample exhibited the best performance in terms of MO discoloration and enhancement of lime carbonation, followed by 0.10FeT and 0.20FeT samples. Further doping with increased Fe content did not contribute to improved performance of TiO_2_ under visible irradiation.

## 4. Conclusions

In the present work, Fe-doped pure anatase phase TiO_2_ nanoparticles were designed with the aim of being used as photocatalysts, and subsequently as admixtures in lime pastes. The undoped and doped TiO_2_ nanoparticles were synthesized through a simple sol-gel method combining hydro-thermal treatment at 75 °C and calcination up to 500 °C. Five different concentrations of Fe, expressed with the Fe content (g) in TiO_2_ (100 g), namely 0.05, 0.10, 0.20, 0.70 and 1.00% w/w, were incorporated into TiO_2_ nanoparticles, with the aim of determining the optimum concentration of the dopant. FTIR revealed the Fe-TiO_2_ formation, whereas XRD evidenced partial transformation from anatase in pure TiO_2_ to brookite in Fe-TiO_2_, as well as decreasing crystallite size as a function of the Fe content. In the UV-Vis analysis, the reduction of the energy band is in a clear relationship with the increasing Fe content, mainly attributed to the charge transfer transition from the Fe d-electrons to the conduction band of TiO_2_. XPS and XANES analyses confirmed the presence of the octahedron structure of TiO_2_ (Ti^4+^) in the anatase crystalline phase and the incorporation of Fe^3+^ into the TiO_2_ crystal lattice. In particular, XANES elucidated maximum deviation from centrosymmetry and average oxidation state equal to Fe^3+^ for the sample 0.10FeT followed by 0.05FeT. These observations were correlated with the better photocatalytic efficiency in degrading the MO under solar radiation with the use of low iron doping titania samples. The structural defects in the lattice give rise to deficiency in oxygen content, which in turn generates polarization fields, thus facilitating the electron-hole separation and enhancing the photocatalytic activity. Finally, the iron doping titania added in pure lime coatings enhanced the carbonation at early stages under solar radiation compared to the undoped titania and pure lime coatings. The most efficient promoter of carbonation proved to be the lowest iron doped titania sample, which also showed considerable photocatalytic efficiency in degrading pollutants, such as MO and MB. The simple and low energy demanding synthesis of 0.05FeT established it as a powerful candidate in the building construction sector, providing easily carbonated lime coatings with self-cleaning properties. 

## Figures and Tables

**Table 1:** Synthesized nanoparticles and lime pastes.

Fe-TiO2 (T) Synthesized Nanoparticles	Pastes of Lime (L) and Fe-TiO2 Nanoparticles (3% w/w)
-	Lime (Blank)
0.00FeT	L0.00FeT
0.05FeT	L0.05FeT
0.10FeT	L0.10FeT
0.20FeT	L0.20FeT
0.70FeT	L0.70FeT
1.00FeT	L1.00FeT

Fe: iron doping; T: TiO_2_; L: Lime.

**Table 2:** Structural data, and textural and optical parameters of Fe-doped TiO_2_ nanoparticles.

TiO2 Nanoparticles	% Anatase	% Brookite	Crystallite Size of Anatase (nm)	Surface Area (m2 g−1)	Band Gap (eV)
0.00FeT	100	-	20.4	52	3.13
0.05FeT	98	2	12.9	65	3.06
0.10FeT	98	2	13.1	76	2.95
0.20FeT	97	3	11.5	n.a.	2.95
0.70FeT	96	4	12.6	n.a.	2.64
1.00FeT	92	8	12.5	59	2.61

* n.a. not available.

**Table 3:** Specific parameters of the photocatalytic efficiency of Fe-doped TiO_2_ nanoparticles.

Sample	k (10−3 min−1)	r2	% Dis. (30 min) *	% Dis. (90 min) *
Blank	0.7	0.97	3	5
0.00FeT	39	0.98	66	98
0.05FeT	44	0.98	71	98
0.10FeT	58	0.96	94	100
0.20FeT	33	0.97	51	100
0.70FeT	8	0.98	16	69
1.00FeT	5	0.99	15	36

* Dis: discoloration of Methyl Orange solution.
